# A novel way to identify specific powdery mildew resistance genes in hybrid barley cultivars

**DOI:** 10.1038/s41598-020-75978-7

**Published:** 2020-11-03

**Authors:** Antonín Dreiseitl

**Affiliations:** Department of Integrated Plant Protection, Agrotest Fyto Ltd, Kroměříž, Czech Republic

**Keywords:** Genetics, Microbiology, Plant sciences

## Abstract

Powdery mildew, a common cereal disease caused by the fungus *Blumeria graminis*, is a major limiting factor of barley production and genetic resistance is the most appropriate protection against it. To aid the breeding of new cultivars and their marketing, resistance genes can be postulated in homogeneous accessions. Although hybrid cultivars (F_1_) should be homogeneous, they are often not genetically uniform, especially if more than two genotypes are involved in their seed production or due to undesirable self-pollination, out-crossing and mechanical admixtures. To overcome these problems the accepted method of postulating specific resistance genes based on comparing response type arrays (RTAs) of genetically homogeneous cultivars with RTAs of standard genotypes was substituted by analysing the frequency of response types to clusters of pathogen isolates in segregating F_2_ generations. This method combines a genetic and phytopathological approach for identifying resistance genes. To assess its applicability six hybrid cultivars were screened and from three to seven with a total of 14 resistance genes were found. Two genes were newly located at the *Mla* locus and their heritability determined. In addition, three unknown dominant genes were detected. This novel, comprehensive and efficient method to identifying resistance genes in hybrid cultivars can also be applied in other cereals and crops.

## Introduction

As the world’s population continues to grow, a consistent supply of highly nutritious food remains a priority. Traditionally-bred cultivars have a limited yield potential to keep pace with population increases. An alternative means to improve crop varieties is the breeding of hybrid (F_1_ generation) cultivars. Barley (*Hordeum vulgare* L.) was the first of the small grain crops from which a commercial F_1_ hybrid barley was marketed half a century ago. However, its potential has not been realised since hybrid breeding research of autogamous cereals has been largely neglected^1^ and yield increases are not enough to offset the higher cost of seed. As new knowledge and techniques are quickly developing this negative situation could rapidly change.

Barley and wheat genomes have already been well-characterised^2,3^ and can be readily modified with CRISPR techniques^4^. This method could be used to select suitable parents for making crosses between those with high combining ability and achieve higher and more stable yields^5,6^. Another recent advance in breeding technology, apomixis, could also be adopted to fix hybrid heterosis in subsequent generations^7^. Hence, hybrid seeds will be easily and cheaply produced and hybrid cultivars can be grown extensively.

Powdery mildew caused by the airborne ascomycete fungus *Blumeria graminis* (DC.) E. O. Speer, f. sp. *hordei* (*Bgh*) emend. É. J. Marchal (anamorph *Oidium monilioides* Link) is one of the commonest diseases of barley^8,9^. The pathogen can be effectively controlled by means of genetic resistance, which is inexpensive and environmentally friendly^10^. *Bgh*, mainly in Europe, is extremely diverse^11–13^ and it is possible to use selected isolates to characterise specific resistance genes and their combinations^14,15^. Several new resistances found by such phenotyping have been postulated in cultivars^16–20^ and more frequently in wild barley, *H*. *vulgare* subsp. *spontaneum*^21,22^.

Specific resistance genes, both singly or in combinations, have little importance for durability of resistance to powdery mildew in the field^9,23,24^. Despite this, a knowledge of specific resistances present in varieties has wide utilization in further research and practise. Considering varietal resistance itself, selected cultivars carrying resistance genes, including those present in hybrid cultivars, can be used as differentials for studying pathogen populations and combined with virulence frequency data. Such results can help to evaluate the effectiveness of individual genes and predict their, usually short, durability as well as the contribution of hybrids on the evolution of pathogen populations. Almost all current barley cultivars contain one or more specific genes^25,26^ and when looking for partial resistance^27–29^ specific genes that mask minor resistance genes must be overcome with corresponding virulent isolates of the pathogen. Furthermore, details of the specific resistances in cultivars is a valuable tool to establish their pedigrees, purity and authenticity^30^.

Identification of resistance genes by postulation is based on obtaining resistance type arrays (RTAs) of varieties and comparing them with RTAs of standard cultivars possessing known resistance genes^31,32^, and to do this successfully requires genetically homogeneous seed^22^. Hybrid cultivars (F_1_ generation) should theoretically be homogeneous, but for various reasons the reality is different. The aim of this research was to develop a new method for identification of specific resistance genes in hybrid crops and to identify powdery mildew resistance genes in a set of six hybrid barley cultivars.

## Results

To accomplish the aim of this research it is necessary to present comprehensive results of the resistance tests. Each of the six tables relating to the screened cultivars has 55 columns and ca. 40–80 lines and these tables are presented in Supplementary Dataset S_1.

The observed numbers of resistant vs susceptible phenotypes (i.e. avirulence (Avir) vs virulence (Vir), respectively) often did not conform to the expected segregation ratios for the number and type of resistance genes. Suggested explanations are given in the text. Data relating to the number and identity of resistance genes and their mode of inheritance are provided in Table 1.

**Table 1:** Fourteen dominant powdery mildew resistance genes identified in six hybrid winter barley cultivars.

Cultivar	Alternative designation	*Ml* resistance genes
Galation		*a1*, *aAl2*, *a8*, *h*, *ra*, (*Ru2*)
Hedy		*aLo*, *ra*, (*IM9*)
Hobbit	NFC 206–26	*a1*, *aAl2*, *a6*, *a14*, *h*, (*u1*), (*u3*)
Mercurioo		*a6*, *a8*, *h*, *aLo*, *ra*, (*u1*), (*u2*)
SU Hylona	DEH 13/1807	*a1*, *aAl2*, *aLv*^a^
Wootan	SY 210–77	*a6*, *a8*, *h*, *ra*, (*u1*), (*u2*)

^a^*MlaLv* is semidominant.

### Galation

Resistance tests of this cultivar were done on one leaf segment grown from 25 ears derived from separate plants and subsequently on five segments from a further 25 ears, making a total of 150 leaf segments individually inoculated with each of 52 isolates. After inoculation with a cluster of 11 isolates (Supplementary Dataset S_1, ordinal numbers 14–24) Avir only to gene *Mla1*, 25–41 (16.7–27.3%) susceptible segments (SSs) were found and proved the expression of a single dominant gene, *Mla1*. Four isolates (27–30) were Avir only to *Ml*(*Ru2*), but after inoculation 172 resistant segments (RSs) (28.7%) were observed, less than expected for a single dominant gene (75.0%). A possibility is that *Ml*(*Ru2*) was heterogeneous in a parent.

Six isolates (ordinal numbers 5–10) were Avir to *Mla1* and *Ml*(*Ru2*) resulting in 13.8% SSs after inoculation, amounting to 6.9% fewer than *Mla1* alone. This also suggests the presence of *Ml*(*Ru2*) in approximately half the frequency perhaps because only one of the lines of a parent possessed it in homozygous form.

Four isolates (3, 4, 13 and 31) were Avir to *Mlra*, but each of them was also Avir to other gene(s). After inoculation with isolate EmA30 (13), which is also Avir to *Mla1*, there were 12.7% SSs, which is fewer than expected with *Mla1* alone, but more than expected for *Mla1* and *Mlra* combined. Hence, *Mlra*, like *Ml*(*Ru2*), might be heterogeneous in one parent.

Isolates 65 and 4–20 (3 and 4) were Avir to *Mlra* as well as to *Mla1* and *Ml*(*Ru2*); 15.3% SSs were recorded after inoculation, indicating that *Mla1* and *Ml*(*Ru2*) were found in “Galation”. However, there was a substantial reduction in the ratio of moderately resistant phenotypes (RTs 1–2, 2 and 2–3), which is typical for *Ml*(*Ru2*), and an increased ratio of highly resistant phenotypes (RTs 0, 0–1 and 1) characteristic of *Mlra*. *Mlra* is localised on chromosome 1H, while the location of *Ml*(*Ru2*) is unknown. These genes often occurred together and *Ml*(*Ru2*) was masked by the highly resistant phenotype *Mlra*. Thus, both *Ml*(*Ru2*) and *Mlra* might be heterogeneous in one parent.

Three isolates (32–34) were Avir only to *Mlh* and 28.2% SSs were recorded after screening, which corresponds to the expression of a single dominant gene, *Mlh*. Two isolates (11 and 12) were Avir to *Mlh* and *Mla1* and testing revealed 4.7% SSs, which confirms both these dominant genes in “Galation”.

Only after inoculation with Race I (26), which was the sole isolate Avir to *Mla8*, were no SSs recovered. This is evidence for the presence of *Mla8* along with a second allelic gene. However, this second gene detected with Race I cannot be the allele *Mla1* since Race I is virulent to it, but must be the closely linked (coupling) pseudo-allele *MlaAl2*. Thus, one of these homozygous genes (*Mla8* or *MlaAl2*) was always present in each of the parents.

In the case of the 18 remaining isolates (35–52) RSs were absent or rare, which could be explained by a mechanical admixture of other genotypes, but do not indicate the existence of any other resistance gene(s) in “Galation”.

### Hedy

Five leaf segments of plants derived from 32 ears were tested. After inoculation with 27 isolates, a total of 2001 RSs (46.3%) were found. Inoculation with 19 isolates (34–52) resulted in susceptibility (RTs 3, 3–4 and 4) of almost all 3040 tested segments with the exception of two RSs arising from a possible mechanical admixture. The results of both clusters of isolates prove the presence of *Ml*(*IM9*) in one of the parents, which was heterogeneous.

After inoculation with four isolates (4–20, 65, D-120 and EmA30; ordinal numbers 28–31), which are Avir to *Ml*(*IM9*) and *Mlra*, 421 RSs (65.8%) were recorded. This is 19.5% more than after inoculation with isolates Avir only to *Ml*(*IM9*) and confirms the presence of *Mlra*, though once again with a lower frequency in the tested offspring.

Isolates J-462 and Race I (32 and 33) were the only ones Avir to *Ml*(*Lo*) typified by very high resistance (RT0) and also Avir to *Ml*(*IM9*), which is characterised by moderate resistance (RT2), respectively. All 320 inoculated segments expressed RT0 providing evidence for the presence of *Ml*(*Lo*) in all tested segments and thus in all parents of Hedy. The recorded RTAs also show that at least 10 out of 32 tested ears contained *Ml*(*Lo*) only.

The above 19 isolates that caused susceptibility throughout the tests indicate that Hedy has no other resistance gene.

### Mercurioo

A total of 150 segments were tested comprising one segment from each of 30 ears and five segments from another 24 ears. After inoculation with seven isolates Avir only to *Mla6* (10–16) there were 286 (27.2%) highly resistant RTs. With another isolate J-462 (20), which is Avir only to *Ml*(*Lo*), 58.7% RSs were observed. Race I (17) is Avir to *Mla6* and *Ml*(*Lo*) and after inoculation only RSs with RTs of 0 and 0–1 were recorded and indicate the presence of dominant gene(s). To explain these differences another allelic gene that is ineffective against any of the other 51 isolates must be considered. This only fits *Mla8* and it is likely that the three alleles *Mla6*, *Mla8* and *Ml*(*Lo*) inherited from at least three parents, are in “Mercurioo”.

Isolate C-132 (19) is Avir only to *Mlh* and the tests revealed 78 RSs (52.0%), which is evidence that one of the parents was heterogeneous for *Mlh*. Six isolates (1–6) were Avir to *Mla6* and *Mlh* and after omitting I-162 (6) because of unusual and inexplicable RTs, a total of 567 (75.6%) RSs were found. Isolates 4–20 and 65 (8 and 9) were Avir to *Mla6* and *Mlra* and inoculations resulted in 159 highly resistant RSs (53.0%). Results after inoculation with both clusters of isolates confirmed the presence of *Mlh* and *Mlra*, though once again in both cases with a lower frequency than expected probably because of parental heterogeneity.

Isolate EmA30 (18) is Avir to *Mlra* and also to *Mla14*, which is often closely linked with *Mla6*. However, after inoculation with this isolate, there were only 58 (38.7%) RSs confirming the presence and absence of *Mlra* and *Mla14*, respectively, in “Mercurioo”.

Tests with isolate PF-512 (21) revealed 119 RSs (79.3%) conferred by an unknown dominant gene given a provisional designation *Ml*(*u1*) and effective only to this isolate.

After inoculation with the remaining 31 isolates virulent to all genes so far detected in “Mercurioo”, many showed a high number of RSs predominantly characterised by RTs of 1–2 to 2–3. Apart from the possibility of there being mechanical admixtures, this indicates the presence of another unknown gene (*Ml*(*u2*)) conditioning moderate resistance. However, since the proportion of RSs was low it is probable that one of the parents was heterogeneous for this allele.

### SU Hylona

One segment from each of forty ears was tested. Inoculation with the first 47 isolates resulted in high proportions of RSs, while the remaining five isolates induced a susceptible response in all except 13 segments which probably resulted from seed impurity. These virulence/avirulence scores are indicative of *Ml*(*Lv*).

After inoculating with isolates 1–13, RSs were exclusively observed and for isolates 14–24 there were only occasional SSs. These 24 isolates are avirulent to *Mla1*. Hence, *Ml*(*Lv*) must be accompanied by *Mla1* and both genes must be allelic. However, in the case of Race I (order number 25), since the isolate is virulent to *Mla1*, a closely linked allele *MlaAl2* is also present in Hylona. After inoculation with 17 (26–42) out of 22 isolates Avir only to *Ml*(*Lv*), segregation ratios conformed to the ratio expected for a single dominant gene. Since there were two distinctly different phenotypes, namely high and moderately resistant, the inheritance of *Ml(Lv)* must be semidominant.

### Wootan

Five plants were taken from each of 24 ears making a total of 120 leaf segments for testing. After inoculation with seven isolates (11–17) Avir only to *Mla6* (RTs 0, 0–1 and 1), 506 (60.2%) RSs were found establishing the presence of this gene albeit recorded in smaller than expected proportions of the offspring. Confirmation of *Mla6* was verified by RTAs recorded on some of the tested ears.

Race I (10) is the only isolate Avir to both *Mla6* and *Mla8* and 119 RSs were observed confirming both these alleles. Because the number of segments with *Mla6* was lower than expected it is likely that there must be a higher number of plants with *Mla8* in the offspring.

After inoculation with isolate C-132 (18), which is the only one Vir to just *Mlh*, 35 SSs (29.2%) were found, while inoculation with six isolates (2–7) Avir both to *Mla6* and *Mlh* resulted in 59 (8.2%) SSs proving that Wootan contains both dominant genes.

The only isolate Avir solely to *Mlra* is EmA30 (19), which gave 73 RSs (60.8%), fewer than expected to confirm the expression of one dominant gene. EmA30 is also Avir to *Mla14*, which is closely linked to *Mla6* in some genotypes, however, the proportion of RSs does not establish resistance involving two genes. Furthermore, the number of RTs showing high resistance is more typical for *Mlra* combined with the absence *Mla14* since the latter is characterised by moderately resistant RTs.

Inoculating with isolates 65 and 4–20 (8 and 9) Avir to *Mla6* and *Mlra*, resulted in 29 SSs (12.1%) indicative of two dominant genes.

Inoculation with isolate PF-512 (20), which is Vir to all four genes detected so far, gave 90 RSs (75.0%) corresponding to a dominant gene identical with one of the unknown genes found in “Mercurioo” (*Ml*(*u1*)).

Testing with many of the remaining 32 isolates often showed RTs with moderate resistance indicative of an unknown gene probably identical to *Ml*(*u2*) in “Mercurioo”, but heterogeneous in one of the parents. This result can also partly be attributed to genotype admixtures.

### Hobbit

Hobbit was the only cultivar studied in the F_6_ generation; one leaf segment derived from each of 60 ears was tested with 52 isolates. After inoculation with 13 isolates Avir to *Mla1* (12–24) there were 396 RSs (50.8%) and shows the presence of *Mla1*.

After inoculation with four isolates Avir to *Mla6* (29–32), 141 RSs (58.8%) were found and confirms this gene too.

Inoculation with nine isolates (1–9) Avir to *Mla1* and *Mla6* (four of which are also Avir to *Mlh*), produced only RSs typical for both these alleles and signifies that they were present in all the offspring. The total proportion of RSs was 9.6% greater than expected (100.0%) and can probably be explained by residual heterozygosity for the two genes that conferred resistance to both clusters of isolates and that were, therefore, counted twice.

Screening with isolate EmA30 (10) also resulted exclusively in RSs with the exception of one SS. EmA30 is Vir to *Mla6* and Avir to *Mla1* and *Mla14*; the latter is closely linked with *Mla6*. Hence, using EmA30 demonstrated that Hobbit possesses *Mla1* and a pseudoallele of *Mla14*.

There were no SSs after screening with Race I (25) Avir to *Mla6* and Vir to *Mla1*. As in the case of *Mla6* and *Mla14*, *Mla1* is also accompanied by the closely linked gene *MlaAl2* to which Race I is Avir and resulted in 15 moderately resistant RTs.

After screening with isolate C-132 (33) that is uniquely Avir just to *Mlh* there were 33 (55.0%) RSs and establishes that Hobbit contains this gene.

Three isolates are Avir to *Mlh* and *Mla6* (26–28) and 73.9% RSs were recorded after testing showing that both these independent dominant genes were in the F_6_ generation of Hobbit.

Among the first 24 isolates that are Avir to *Mla1* only PF-512 (11) is Avir to *Ml*(*u1*). After inoculation with this isolate 46 RSs (76.7%) were observed and verifies the presence of *Mla1* and *Ml*(*u1*) in this cultivar.

After testing with 17 of the remaining 19 isolates (34–50) there were sometimes low frequencies of RSs (in total 25) probably as a result of mechanical admixtures in the seed stocks. Nevertheless, inoculation with isolates J-462 and Y-4 resulted in 37 RSs (30.8%) suggesting an unknown gene (*Ml*(*u3*)) conditioning moderate resistance and present in one of the two lines of one parent.

## Discussion

Hobbit was the first hybrid cultivar among a large group of candidates for registration in the Czech Republic. Its first resistance tests were done in 2011 when it was entered into second year registration trials. However, the information that Hobbit is a hybrid cultivar was not known to us at the time, nor for Wootan that was included in trials two years later. Despite repeated testing, the resistance genes in the F_1_ generation of these two cultivars were not identified (results are not shown) because RTAs did not correspond to known resistances due to the presence of allelic genes, of heterogeneous RTs^26^ and the unavailability of pedigrees. The other four cultivars were already known to be hybrids and they were no longer tested as F_1_s.

The F_2_ generation was, therefore, used for further tests of five out of the six cultivars and only Hobbit was successively sown to obtain F_6_ seed to generate homogeneous offspring for gene postulation^31,33^. Nevertheless, this was not achieved, primarily because there were seven genes, which were identified later, and also that out-crossing and mechanical admixtures might have occurred. Hence, it is preferable that even for the F_6_ generation it was better to analyse the frequency of RTs rather than focus on RTAs of individual offspring. The demanding and time-consuming successive sowings for later generation seed production was inefficient and unsuitable for obtaining homogeneous RTAs of Hobbit and it cannot be recommended for winter barley cultivars which generally have numerous specific resistance genes^26^.

Pedigrees of cultivars give some indication of which genes are likely to be present, and tests and analyses can verify the presence or absence of genes known to be in the parents^34^. However, breeding companies did not provide this information for most of the hybrid cultivars, and the one that was supplied for SU Hylona (CMS04LM183L001 × 10HR170D002) was not useful as a starting point for identifying resistance genes.

Heterogeneity of hybrid cultivars (F_1_ generation) can be caused by more than two genotypes involved in their formation, for example a male sterile female parent, a fertility restorer male parent and fertile maintainer^6^. Heterogeneity may also result from self-pollination if the cytoplasmic male sterility becomes unstable through environmental factors including temperature^35^. Second, some parental cultivars may consist of more than one line. Third, heterogeneity may be caused by out-pollination with different cultivars grown nearby, and fourth, there may be mechanical seed admixtures with other genotypes^30^. These factors could seriously affect the ratios of phenotypes, which may differ from the expected ratios. Some of the above reasons could explain why, for instance, in 2019/2020 a breeding company stated that only 80% of seeds for one of their winter barley candidates for registration were actually hybrid. Incomplete dominance of resistances, such as *MlLv* in SU Hylona, can also cause phenotypic heterogeneity in hybrid cultivars.

Ratios of resistant/susceptible plants are affected by the location of genes too. In the six cultivars tested, 14 specific powdery mildew resistance genes were found, six of which, including *Mlra*, are located on chromosome 1H. Three of these are localised at the *Mla* locus (*Mla1*, *Mla6* and *Mla8*) and two are closely linked with this locus (*Mla14* and *MlaAl2*)^36^. The results of this study show that *MlLo* and *MlLv*^17,18^ identified in three of the cultivars, are also localised in the *Mla* locus or are tightly linked to it. *Mlh* is located on chromosome 6H, while the location of *Ml*(*IM9*) and *Ml*(*Ru2*) as well as three detected unknown genes has not been reported.

Our testing revealed from three to seven resistance genes in each of the six hybrid cultivars, with a total frequency of 32. The commonest genes were *Mlh* and *Mlra* found in four cultivars. *Mla1, Mla6*, *Mla8* and *Ml*(*u1*) were found in three cultivars; *Mla1* closely linked to *MlaAl2* in each case, whereas *Mla6* was closely linked with *Mla14* in only one of them. In addition, *MlLo* and *Ml*(*u2*) were present in two cultivars and *Ml*(*IM9*), *MlLv*, *Ml*(*Ru2*) and *Ml*(*u3*) in one cultivar. *Ml* genes *h, ra*, *Lo,* (*IM9*) and *Lv* are common in winter barley cultivars, while *a1, a6* and *a8* are typical in spring barleys^37^, two of which, *a1* and *a6*, are often accompanied by "additional" genes *MlaAl2* and *Mla14*, respectively^36^. *Mla6* is currently one of the most frequent genes found in winter cultivars too^26^.

Basic requirements for resistance to plant pathogens are effectiveness and durability. Monogenic specific resistances, particularly those newly-introduced, are often characterised by initial high effectiveness to almost all pathotypes that appear in the given area and often exhibit a low RT (0, 0–1 or 1) that does not allow even limited reproduction of the pathogen. However, *Bgh* is extraordinarily adaptable^23^; its population is usually large and frequent mutations from avirulence to virulence or migration of virulent pathotypes from other areas often occur. Then, cultivars with corresponding resistance genes cause directional selection of new pathotypes that can rapidly reproduce leading to sudden collapse of the resistance in the field.

The pathogen isolates used for resistance tests were virulent to all genes present in the six cultivars. Recent studies of central European populations of *Bgh* also revealed a high frequency of virulence to all genes identified here^13^. Thus, it can be concluded that even the high complexity of common resistance genes is not an important contributor to cultivar resistance in field conditions and the practical effect of these combinations is, therefore, negligible.

Limited durability of specific resistances can be extended by ‘pyramiding’ fully effective resistances into one genotype^38^. In this instance such resistances should not be used individually in other cultivars so that they are not quickly overcome and do not render the gene pyramids ineffective although this restriction is hard to implement in practice.

Another option for extending limited durability of specific resistances that can be used only in hybrid cultivars is combining fully effective resistances conferred by dominant genes at the same locus^39^. Jørgensen^36^ published an overview of several dozen alleles localised in, or closely linked with the *Mla* locus. The author did not include all known genes at *Mla*^40^ and many others have been found subsequently^41^, including three newest^42–44^ and two which were localised herein. Seven alleles or pseudoalleles localised in the *Mla* locus or closely linked with it were found here, but in every case these were genes against which there is already a high frequency of virulences in Europe^13,45^. The option to extend the effectiveness of new specific resistances located at the same locus available only in hybrid cultivars, was not utilized in any of the tested cultivars, but would be of practical benefit in the future hybrids.

Our initial effort for gene identification aimed to obtain RTAs by testing plant progenies harvested from individual ears. However, this led to limited success only where resistance genes were individually present and was rarely encountered in cultivars containing from three to seven genes. Therefore, RTAs were substituted with clusters of results (RT frequencies) after inoculation with isolates grouped according of their virulence/avirulence arrays. A good example of this can be seen particularly in SU Hylona, which contains *MlLv*. This new method does not necessitate individual plant progeny testing because a bulk of seed produced from a hybrid cultivar is suitable for testing and less time consuming.

The genetic approach to the identification of resistance genes is based on analysing genotypic ratios, mainly in a segregating F_2_ generation, while the phytopathological approach is based on postulating these genes, i.e. comparing the RTA of a given cultivar with RTAs of standard genotypes. The present research successfully combined both these approaches and can be applied to identifying resistance genes against numerous pathogens in barley and other crop species.

## Conclusions

- The aim of this research was to postulate resistance genes against powdery mildew in a set of commercial hybrid barley cultivars
- The basic pre-requisite for resistance gene postulation is homogeneity of tested genotypes
- For resistance gene postulation, seed of F_1_ hybrids has been tested with a set of pathogen isolates
- Despite repeated testing the aim was not achieved, first, because of unexpected heterogeneity of response types found within cultivar–isolate interactions; second, response type arrays did not correspond to the response type arrays of known resistance genes due to the presence of allelic genes; third, phenotypes of some genes were masked by the phenotypes of other genes, and fourth, because the pedigree of the hybrids was unknown
- A new method that generally combines genetic (genetic analysis) and phytopathological (resistance gene postulation) approaches has been developed
- The screening of F_1_ generations was substituted with segregating F_2_ populations that were tested with a large set of isolates
- Genetic analysis of evaluated response types of F_2_ progenies established the number of resistance genes that were present in interactions between hybrid cultivar and pathogen isolate, their inheritance and possible allelism
- The total number of genes present in a hybrid cultivar and their identity have been determined in a similar way to that of gene postulation when response type arrays were substituted with groups of the pathogen isolates clustered according to their virulence/avirulence to individual resistance genes
- This new method has fulfilled the aim and has been verified by testing with six hybrid cultivars; a wide range of results confirms its performance
- This method is suitable for identifying specific resistances in many hybrid crops.

## Methods

### Plant material and pathogen isolates

Six hybrid winter barley cultivars were screened for their response to powdery mildew. F_1_ and F_2_ generations of Wootan were tested as well as the F_1_ and F_6_ generations of Hobbit and the F_2_ generations of the remaining four cultivars (Galation, Mercurioo, Hedy and SU Hylona). F_1_ seed was obtained from authorities conducting the Czech registration trials. Separately harvested ears of the F_2_ generation were collected from yield trials and propagated plants of Hobbit (F_6_ generation) grown at the Agricultural Research Institute Kromeriz.

F_1_ generations were inoculated with 51 to 58 selected *Bgh* reference isolates, while for tests of the F_2_ generations of five cultivars and the F_6_ generation of Hobbit 52 isolates were used. The isolates had previously been collected in 14 countries in all nonpolar continents over a period of 65 years (1953–2018) and represented the global virulence/avirulence diversity of the pathogen. Before inoculation, all isolates were checked for their purity and their correct pathogenicity phenotypes verified on standard barley lines^46^. The isolates were multiplied on leaf segments of susceptible cultivar Stirling. The ordinal number of an isolate may differ on each sheet of Supplementary Dataset S_1 depending on the sorting of isolates by their virulence/avirulence arrays to identified resistance genes.

### Resistance tests and evaluation

Approximately 15, 30 or 50 seeds of each ear were sown in pots (80 mm diameter) filled with a gardening peat substrate and placed in a mildew-proof greenhouse under natural daylight. Leaf segments 15 mm long were cut from the central parts of healthy fully-expanded primary leaves when second leaves were emerging. Three segments for the F_1_ generation and one or five segments for the F_2_ and F_6_ generations were placed adjacently along with four segments of the susceptible Stirling oriented diagonally and with their adaxial surfaces facing upward in a 150 mm Petri dish on water agar (0.8%) containing benzimidazole (40 mg^−L^), a leaf senescence inhibitor^20^. Over a period of several years, 18–27 leaf segments of each cultivar in the F_1_ generation were tested with each isolate (results are not shown), while 120–160 segments (only 40 for SU Hylona) were tested in the F_2_ generation and 60 segments were tested for the F_6_ generation of Hobbit.

Seven days after inoculation, phenotypes of barley cultivar x pathogen isolate interactions equating to response types (RTs) were scored on the central part of the adaxial side of leaf segments. For evaluation, a nine point scale ranging from 0–4 including intertypes was used, where 0 = no visible mycelium or sporulation, and 4 = strong mycelial growth and sporulation on the leaf segment^47^. One day later the scoring was repeated and considerable differences were re-assessed. Six RTs (0 to 2–3) were considered resistant whereas RTs 3, 3–4 and 4 were susceptible. A set of RTs to all isolates provided a response type array (RTA) for each cultivar but since RTAs in the segregating populations were often heterogeneous the isolates were grouped into clusters according to virulence/avirulence frequencies. Based on the gene-for-gene model the resistance genes were identified by comparing the isolate clusters with RTAs of standard barley genotypes possessing known resistance genes. χ^2^ tests were used to assess whether the observed resistance/susceptibility ratio for one or two dominant genes deviated (p < 0.05) from the expected ratio, 3:1 for one gene and 15:1 for two genes because homozygous and heterozygous resistant plants could not be distinguished phenotypically and only resistant and susceptible plants could be identified.

## Supplementary information

Supplementary Information 1.

Supplementary Information 2.

## Data Availability

Materials, data and associated protocols are available to readers. All data generated and analysed during this study are included in this article and it’s a Supplementary dataset file.
