# Supplementary material for: A novel way to identify specific powdery mildew resistance genes in hybrid barley cultivars
Source: Sci Rep. 2020 Nov 3;10:18930. doi: 10.1038/s41598-020-75978-7 (PMC7641246; doi:10.1038/s41598-020-75978-7)
Supplement: Supplementary file 2 — Supplementary Information 2. [file 41598_2020_75978_MOESM2_ESM.doc]

*Manuscript title*

**A novel way to identify specific powdery mildew resistance genes in hybrid barley cultivars**

*Author list*

Antonín Dreiseitl
